# Case Report: Clinical Features of a Chinese Boy With Epileptic Seizures and Intellectual Disabilities Who Carries a Truncated *NUS1* Variant

**DOI:** 10.3389/fped.2021.725231

**Published:** 2021-08-31

**Authors:** Pingli Zhang, Di Cui, Peiyuan Liao, Xiang Yuan, Nuan Yang, Yuanyuan Zhen, Jing Yang, Qikun Huang

**Affiliations:** ^1^Department of Pediatrics, Qilu Hospital (Qingdao), Cheeloo College of Medicine, Shangdong University, Qingdao, China; ^2^Running Gene Inc., Beijing, China

**Keywords:** whole-exome sequencing, *NUS1*, epilepsy, MRD55, ID

## Abstract

The mental retardation-55 with seizures (MRD55) is a rare genetic disease characterized by developmental delay, intellectual disability, language delay and multiple types of epileptic seizures. It is caused by pathogenic variants of the *NUS1* gene, which encodes Nogo-B receptor (NgBR), a necessary subunit for the glycosylation reactions in mammals. To date, 25 disease-causing mutations of *NUS1* have been reported, which are responsible for various diseases, including dystonia, Parkinson's disease, developmental and epileptic encephalopathy as well as congenital disorder of glycosylation. In addition, only 9 of these mutations were reported with detailed clinical features. There are no reports about Chinese cases with MRD55. In this study, a novel, *de novo* pathogenic variant of *NUS1* (c.51_54delTCTG, p.L18Tfs^*^31) was identified in a Chinese patient with intellectual disability and epileptic seizures. This pathogenic variant resulted in truncated NgBR proteins, which might be the cause of the clinical features of the patient. Oxcarbazepine was an effective treatment for improving speech and movement of the patient, who consequently presented with no seizure. With this novel pathogenic variant found in *NUS1*, we expand the genotype spectrum of MRD55 and provide valuable insights into the potential genotype-phenotype correlation.

## Introduction

The mental retardation-55 with seizures (MRD55, OMIM 617831) is a rare genetic disease characterized by developmental delay, intellectual disability, language delay and multiple types of epileptic seizures (1). It is caused by pathogenic variants of the *NUS1* gene, which encodes Nogo-B receptor (NgBR) (2). NgBR is a conserved subunit of the dehydrodolichyl diphosphate synthase (DDS) complex, which promotes cisprenyltransferase (cis-PTase) activity and is necessary for the glycosylation reactions in mammals (3). To date, 25 disease-causing mutations of *NUS1* have been reported (according to the Human Mutation Database), nine of which were reported with detailed clinical phenotypes. These nine pathogenic variants, however, are associated with four various diseases, including dystonia (4), Parkinson's disease (5, 6), developmental and epileptic encephalopathy (1, 6–8), and congenital disorder of glycosylation (3). Thus, it seems that different pathogenic variants cause distinct diseases. In addition, cases with genotypes and detailed clinical features are limited. Additional cases are needed for a better understanding of the relationship between MRD55 and the associated pathogenic variants.

Here, we report the clinical and molecular characterization of the first Chinese MRD55 patient, who displayed intellectual disability and epileptic seizures, and had a novel, *de novo* pathogenic variant of *NUS1* (c.51_54delTCTG, p.L18Tfs^*^31). With this novel pathogenic variant found in *NUS1*, we expanded the genotype spectrum of MRD55.

## Methods

### Case Presentation

Our patient was the first child to non-consanguineous, healthy parents. He was born full-term *via* cesarean delivery, G1P1, with no asphyxia. The birth parameters included weight 3.0 kg (Z = −0.925), length 50.0 cm (Z = 0.004), and head circumference (HC) 38.0 cm (Z = 1.299). There was no abnormality in the perinatal period, and neither of his parents had a related family history. He could roll over, sit, call his parents and walk independently at the ages of 3, 6, 12, and 16 months, respectively. When the patient was 3 years old, he presented with unstable standing, involuntary movement of the limbs, abnormal posture and dance-like movements when he was emotionally agitated or fatigued. During this period, the patient was conscious and occasionally experienced sudden falls or involuntary squatting, with no binocular upward gaze, perioral bruising, or limb stiffness. The whole duration lasted approximately several seconds to several hours, which could be relieved spontaneously after the patient rested. The results of brain magnetic resonance imaging (MRI) were normal whereas video electroencephalogram (EEG) revealed slow and spiny-slow waves in the bilateral frontopolar, frontal, and anterior temporal regions during the sleeping period. The diagnosis of epilepsy was confirmed, and he was treated with Depakine (6 mg/kg/day) for a month. The treatment was not effective, and the patient continued to have seizures and unsteady standing in a variable frequency. The patient gradually developed occasional instability in the walking gait. He did not have any malformations or autistic feature but presented with a moderate intellectual disability at the age of 4 years and 7 months. The scores of full-scale intelligence quotient (FIQ), performance intelligence quotient (PIQ) and verbal intelligence quotient (VIQ) were 48, 47, and 61, respectively, as tested by Korean-Wechsler Preschool and Primary Scale of Intelligence (K-WPPSI). He was not given any treatment this time. At the age of 6 years, the patient suffered convulsions. The symptoms included unconsciousness, rolled-up left eye, stiff upper limbs and cyanotic lips. The duration of each convulsion was ~5 min, and the patient was admitted to the hospital for “paroxysmal dyskinesias.” The following abnormalities were detected in the video EEG: paroxysmal hypertonic 2–3 Hz slow waves interspersed with spikes were found in each conductor, especially in the frontal region. No other abnormality was identified from the blood or urine test, metabolic profiling analyses (amino-acid and acyl-arnitine profiles) or MRI scans (cervical, thoracic and lumbar spine). The patient was treated with madopar (2.1 mg/kg/day, Q12h) for a month, but did not improve. He still walked unsteadily and sometimes stood unsteadily. The treatment was discontinued. After 15 months, when the patient was 7 years and 3 months old, convulsions occurred during the quiet sleep. The symptoms were similar to those described before. Physical examination was performed, and the results were all normal except for the video EEG. The background activity was slow, with slow wave emitted in the left frontopolar during sleep. Meantime, the Gesell developmental scales were performed, and the results indicated a developmental stage of 2 years old. The patient was given oxcarbazepine (40 mg/kg/d) and sensory integration training. The treatment was effective, and the patient consequently did not have seizures. His speech and movement improved. However, he still presented with an unsteady walking gait, slow and slurred communication and poor numeracy. He was unable to add or subtract within ten digits of recognizable numbers. Additionally, his logic and comprehension were poor.

### Genetic Analysis

The peripheral blood of the patient was collected and sent to Running Gene Inc. (Beijing, China) for whole-exome sequencing (WES) to identify the causal gene. Briefly, DNA was isolated and fragmented to build a DNA library by using the KAPA Library Preparation Kit (Illumina, Inc., USA). Then, the library was sequenced using an Illumina HiSeq X10 platform (Illumina, San Diego, USA) using a 150-bp paired-end reads according to the standard manual. The sequencing data was filtered and aligned with the human reference genome (GRCh37/hg19) by using the BWA Aligner (http://bio-bwa.sourceforge.net/). and variants were annotated by ANNOVAR (annovar.openbioinformatics.org/en/latest/). The candidate causal genes thereby discovered were then confirmed *via* Sanger sequencing.

## Results

**Figure 1:**
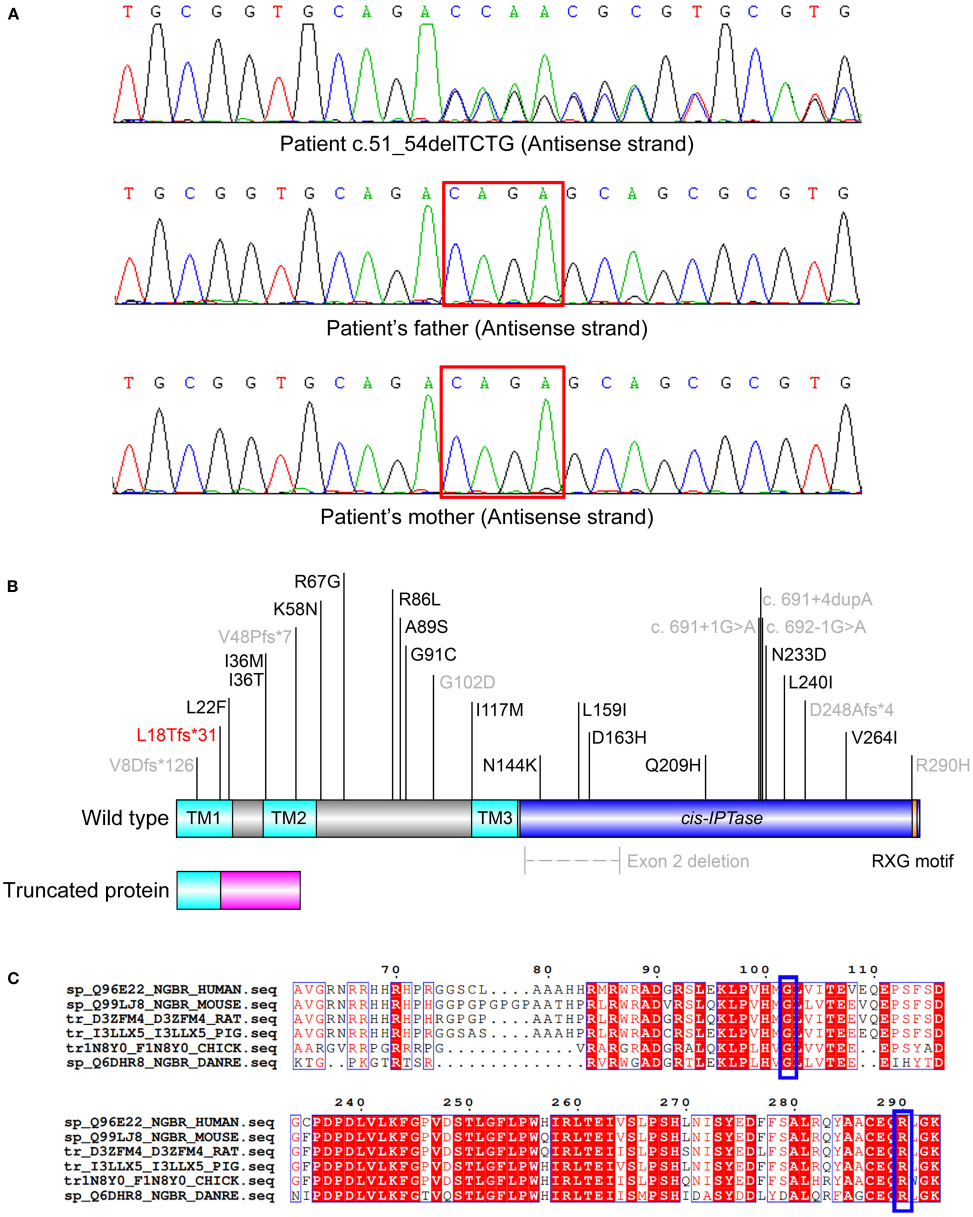
**(A)***De novo* heterozygous variants of c.51_54delTCTG in the patient. **(B)** Scheme of the reported disease-causing mutations of *NUS1*. The transmembrane (TM) 1 (AA 1-23), TM2 (AA 35-56) and TM3 (AA 117-135) domains are colored in cyan. The *cis-IPTase* domain (AA 136-293) is colored in blue. The RXG motif (AA 290-292), which is crucial for the prenyltransferase activity, is colored in orange. The 31 miscoded amino acids of the truncated protein are colored in magenta. The pathogenic variant reported in this study is colored in red and those reported with detailed clinical features are colored in gray. **(C)** Alignment of the NUS1 proteins of various species. Residues G102 and R290 (indicated in blue boxes) are highly conserved.

A novel variant c.51_54delTCTG (p.L18Tfs^*^31) of *NUS1* (NM_138459.3) was identified in the patient *via* WES analysis (Figure 1A). This *de novo* (PS2) heterozygous deletion in exon 1 introduces a premature terminator and results in a truncated protein with 31 miscoded amino acids (Figure 1B). The truncated protein consists of 18 correctly encoded amino acids, constituting part of the transmembrane domain 1 (TM1). Other functional domains (TM2, TM3, the *cis*-IPTase domain and the RXG motif) are expected to be deleted, resulting in the loss of function (PVS1). This vatiant is absent from the controls (1000 Genomes, ExAC, gnomAD, and CNGB) (PM2). Thus, variant c.51_54delTCTG is classified as a pathogenic variant of *NUS1* according to the standard of American College of Medical Genetics (9), and was predicted to cause MRD55. No other pathogenic variant was identified in genes that may be associated with the developmental delay and epileptic encephalopathy of our patient.

## Discussion

MRD55 is a rare congenital disease characterized by developmental delay, intellectual disability, language delay and multiple types of seizures (1). It was first reported in three unrelated patients, who presented with onset of myoclonic seizures and carried heterozygous, *de novo* pathogenic variants of the *NUS1* gene (1). The diagnostic criterion for this disorder were established. Considering the epileptic seizures, moderate intellectual disability and the identified pathogenic *NUS1* variant, the patient was diagnosed with MRD55.

*NUS1* encodes Nogo-B receptor, which consists of 293 amino acids and regulates cis-PTase activity (3). It interacts with the dehydrodolichyl diphosphate synthase complex subunit (DHDDS), forming a biological functional heteromer (the DDS complex). DDS is essential for the biosynthesis of dolichol monophosphate (Dol-P), which is utilized as a sugar carrier during protein glycosylation in the endoplasmic reticulum (ER) (3, 10–12). Therefore, the protein encoded by pathogenic *NUS1* variants might lack the proper function, and thus the regulation of protein glycosylation is disrupted and disease ensue.

To date, 25 disease-causing mutations of *NUS1* have been reported (Figure 1B), nine of which are described with detailed clinical phenotypes (Table 1) (1, 3–8). It is interesting that ten pathogenic variants, including the one reported in our study, are responsible for four different diseases, including dystonia, Parkinson's disease, CDG1AA and MRD55. Phenotypes are diverse, patients present with seizures (13/16), intellectual disability (ID, 12/16), language delay (5/16), developmental delay (6/16), ataxia (10/16), scoliosis (4/16) and hypotonia (3/16). Autism and dysmorphic features are rare, which are only found in one MRD55 (1) and two CDG1AA patients (3), respectively. All the *NUS1* pathogenic variants carried by MRD55 patients are truncated variants (frameshift, splicing and exon deletion), suggested to cause MRD55 features *via* a mechanism of haploinsufficiency (1). Two heterozygous variants have been detected in patients with Parkinson's disease. One had the splicing-site variant c.691+4dupA, which generates an aberrant spliced mRNA, leading to a significant reduction in the NUS1 level (5). The other one carries the frameshift variant c.22_23insA, which is barely expressed in human embryonic kidney (HEK) cells 293 cells, indicating a similar reduction in NUS1 level (6). In the patients with dystonia and CDG1AA, the missense variants p.G102D (heterozygous) and p.R290H (homozygous) were observed, respectively. G102 and R290 are phylogenetically highly conserved (Figure 1C), and their substitutions are expected to impair the function of NUS1.

**Table 1:** Clinical features of individuals with *NUS1* pathogenic variants.

**Origin**	**This study**	**Fraiman et al**.	**Araki et al**.	**Araki et al**.	**Araki et al**.	**Araki et al**.	**Araki et al**.	**Wirth et al**.	**Den et al**.	**Den et al**.	**Guo et al**.	**Hamdan et al**.	**Hamdan et al**.	**Hamdan et al**.	**Park et al**.	**Park et al**.
Individuals	1	2	3	4	5	6	7	8	9	10	11	12	13	14	15	16
Pathogenic variant (ACMG classification)	c.51_54delTCT, G.p.L18Tfs*31 (PVS1+PS2+PM2, Pathogenic)	c.692-1G>A splice (PVS1+PS2+PM2, Pathogenic)	c.22_23insA, p.V8Dfs*126 (PVS1+PM2+PP1, Pathogenic)	c.22_23insA, p.V8Dfs*126 (PVS1+PM2+PP1, Pathogenic)	c.22_23insA, p.V8Dfs*126 (PVS1+PM2+PP1, Pathogenic)	c.22_23insA, p.V8Dfs*126 (PVS1+PM2+PP1, Pathogenic)	c.22_23insA, p.V8Dfs*126 (PVS1+PM2+PP1, Pathogenic)	c.305G>A, p.G102D (PS2+PM2+PP3, Likely pathogenic)	c.691+1G>A, splice (PVS1+PS2+PS3+PM2, Pathogenic)	c.691+1G>A, splice (PVS1+PS2+PS3+PM2, Pathogenic)	c.691+4dupA, splice (PVS1+PS2+PS3+PM2, Pathogenic)	c.743delA, p.D248Afs*4 (PVS1+PS2+PM2, Pathogenic)	c.128_141dup14, p.V48Pfs*7 (PVS1+PS2+PM2, Pathogenic)	exon 2 deletion, 1.3 kb (PVS1+PS2+PM2, Pathogenic)	c.869G>A, p.R290H (PS3+PM2, Likely pathogenic)	c.869G>A, p.R290H (PS3+PM2, Likely pathogenic)
Zygote type/Inheritance	Het/*De novo*	Het/*De novo*	Het/N/A	Het/Paternal	Het/ N/A	Het/maternal	Het/maternal	Het/*De novo*	Het/*De novo*	Het/*De novo*	Het/*De novo*	Het/*De novo*	Het/*De novo*	Het/*De novo*	Hom/Parents	Hom/Parents
Disease	MRD55	MRD55	Parkinson's disease	MRD55 without ID	MRD55	MRD55	MRD55	Dystonia	MRD55	MRD55	Parkinson's disease	MRD55	MRD55	MRD55	CDG1AA	CDG1AA
Gender	Male	Male	Male	Female	Male	Female	Female	Male	Female	Male	Female	Male	Male	Female	Male	Male
Age	7 years 3 months	31 years	77 years	44 years	42 years	17 years	15 years	28 years	17 years	59 years	26 years	8 years 9 months	15 years	29 years	Deceased at 29 months	4 years
Ethnicity	Chinese Korean	N/A	Japanese	Japanese	Japanese	Japanese	Japanese	N/A	Japanese	Japanese	Chinese Han	N/A	French-Canadian	Caucasian	Czechs	Czechs
Family history	–	–	–	Two daughters	N/A	Mother	Mother	–	Elder brother (febrile seizures)	N/A	N/A	N/A	N/A	N/A	Brother (patient 10)	Brother (patient 9)
Birth weight	3.0 kg	N/A	N/A	N/A	N/A	N/A	N/A	N/A	2.826 kg	3.5 kg	N/A	N/A	N/A	N/A	N/A	N/A
Birth length	50.0 cm	N/A	N/A	N/A	N/A	N/A	N/A	N/A	N/A	56.0 cm	N/A	N/A	N/A	N/A	N/A	N/A
Age of onset	3 years	13 years	N/A	N/A	N/A	N/A	N/A	7 years	9 months	8 years	16 years	12 months	10 months	2.5 years	11 months	7 months
Seizures	Atonic seizures, tonic-clonic seizures, focal seizers, tonic seizers,	Tonic-clonic seizures	–	Clonic seizures	Absence seizures, Tonic-clonic seizures	Absence seizures	Absence seizures	–	Febrile seizure at 9 months, generalized tonic-clonic convulsion without fever at 14 months, status epilepticus at 6 years 3 months	Loss of consciousness without convulsion at 8 years	N/A	Generalized myoclonic epilepsy, convulsive epilepsy, nocturnal jerks	Myoclonic absences with behavioral arrest, facial and palpebral myoclonus	Myoclonic absences with behavioral arrest and eyelid flutters, as well as limb myoclonus	Tonic-clonic seizures, refractory epilepsy and recurrent attacks of “status epilepticus”	Refractory epilepsy, severe seizure
Frequency of seizures	in an irregular number	N/A	N/A	N/A	N/A	N/A	N/A	N/A	Seizure-free since 6 years of age	N/A	N/A	N/A	5 times a day, lasting 5–10 s	1–2 times a week	N/A	N/A
EEG	Spiny slow wave and slow wave, paroxysmal hypertonic 2–3 HZ slow waves interspersed with spikes, slow background and slow wave emission	Spikes and polyspikes-waves, sometimes with slow spike-wave complexes	Normal	Normal	Slow rhythm (5–7 Hz), Generalized ~3 Hz spike and wave complexes	Generalized ~3 Hz spike and wave complexes	Slow rhythm (7–8 Hz), Generalized ~3 Hz spike and wave complexes	N/A	3-Hz, diffuse, spike-and-slow-wave, complexed with 7-Hz, slow wave background	8–9 Hz slow α rhythm background with no epileptiform activity	N/A	Bifrontal epileptiform activity	Diffuse background slowing, with rhythmic, bifrontal, high-amplitude theta discharges	Generalized spike-wave and poly-spike wave activity	N/A	N/A
Effective medicines for seizures	Oxcarbazepine	Levetiracetam	Zonisamide, Clonazepam	Valproic acid, Clonazepam	Valproic acid, Clonazepam	Valproic acid	Valproic acid	N/A	Valproic acid was effective for seizures, Normal lessened 3-Hz, diffuse, spike-and-slow wave complexes	Myoclonus lessened with 50 mg baclofen	N/A	Levetiracetam	N/A	Relatively well-controlled with a combination of valproic acid, lamotrigine and clonazepam	N/A	N/A
Brain MRI	Normal	Thickening of the corpus callosum	Cerebellar atrophy	Suspected cerebellar atrophy	Cerebellar atrophy	Suspected cerebellar atrophy	Cerebellar atrophy	Normal	Normal at 20 months, slight cerebellaratrophy at 14 years	Normal	Normal	Normal (2 years 3 months)	Normal (8 years)	Normal	N/A	Severe cortical atrophy
ID	Moderate	Mild to moderate	–	–	Mild	Mild	Moderate	Mild	Mild to moderate	Moderate	N/A	Moderate	Moderate	Mild	Yes	N/A
Language delay	Yes	N/A	N/A	N/A	N/A	N/A	N/A	N/A	Mild (speaking two-word sentences at 2 years)	Yes	-	Yes	Mild	-	N/A	N/A
Developmental delay	Yes	N/A	N/A	N/A	N/A	N/A	N/A	N/A	Mild psychomotor delay	–	N/A	Yes	Yes	Mild motor delay	Yes	N/A
Ataxia	Yes	Mild	Yes	Yes	Yes	Yes	Yes	–	Yes	Yes	N/A	Yes	–	–	N/A	N/A
Autism	–	N/A	N/A	N/A	N/A	N/A	N/A	N/A	–	–	N/A	N/A	Yes	N/A	N/A	N/A
Scoliosis	–	N/A	N/A	N/A	N/A	N/A	N/A	N/A	Yes (operation at 15 years of age)	Yes	N/A	N/A	N/A	N/A	Yes, congenital	Yes, congenital
Hypotonia	–	N/A	N/A	N/A	N/A	N/A	N/A	Segmental non progressive dystonia of upper limbs	–	–	N/A	N/A	N/A	N/A	Severe	Severe
Dysmorphic features	–	N/A	–	–	–	–	–	N/A	–	–	N/A	N/A	–	N/A	Microcephaly	Microcephaly
Additional features	–	Psychosis	Resting and intention tremor, Myoclonus	Resting and intention tremor	Intention tremor	Intention tremor	Resting and intention tremor, Myoclonus	Myoclonus, tremor	Dysgraphia due to tremulous myoclonus of bilateral extremities	Eye pursuits were saccadic, hyperkinesia volitionelle-like movement, cortical myoclonus	Parkinson's disease, asymmetric onset, bradykinesia, resting tremor in limbs, mild gait difficulties	–	–	Eye pursuits were saccadic, but saccades were normal	Histophathological examination of autopsy tissue revealed non-specific neuronal loss in brain cortex and cerebellum	No

*PVS1, null variant (non-sense, frameshift, canonical ±1 or 2 splice sites, initiation codon, single or multi-exon deletion) in a gene where loss of function (LOF) is a known mechanism of disease; PS2, de novo (both maternity and paternity confirmed) in a patient with the disease and no family history; PM2, absent from controls (or at extremely low frequency if recessive) in Exome Sequencing Project, 1000 Genomes or ExAC; PP1. Co-segregation with disease in multiple affected family members in a gene definitively known to cause the disease; PP3, Multiple lines of computational evidence support a deleterious effect on the gene or gene product; MRD55, mental retardation-55 with seizures; CDG1AA, congenital disorder of glycosylation type Iaa; ID, intellectual disability; N/A Not available, Not assessed; Het, Heterozygous; Hom, Homozygous*.

**Figure 2:**
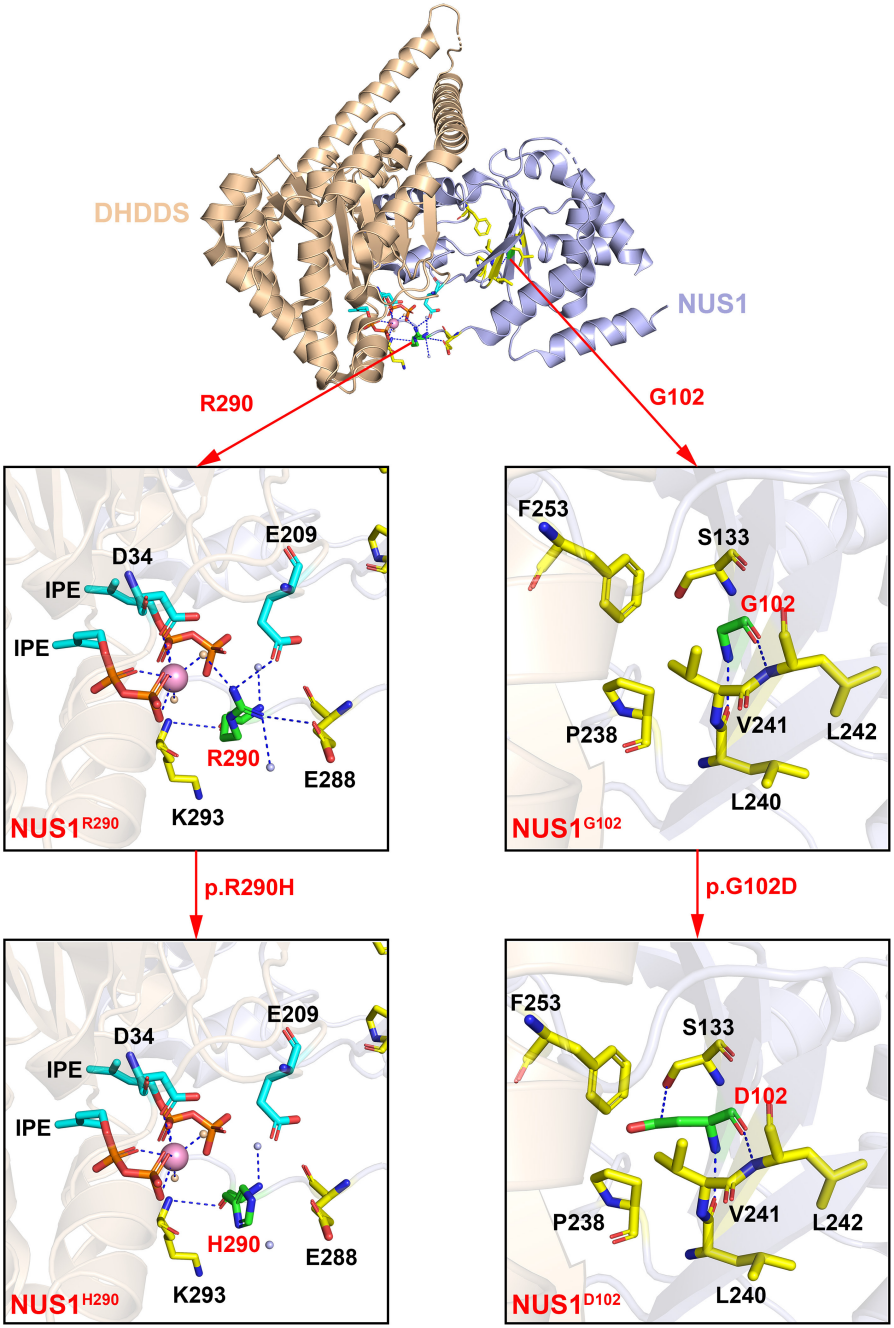
The crystal structure of the human NgBR/DHDDS complex (PDB code: 6W2L). Shown are the cartoon representation of the wildtype NgBR/DHDDS structure and the G102, R290 mutants. The DHDDS and NgBR subunits of the heterodimer are colored in wheat and light blue, respectively. Residues G102, R290 and their pathogenic variants D102, H290 are shown in green sticks. D34, S133, E209, P238, L240, V241, L242, F253, E288, E293 and two IPEs are shown in sticks and highlighted in cyan (DHDDS subunit) and yellow (NgBR subunit). The water molecules of DHDDS and NgBR are colored in orange and light blue, respectively. The metal ion Mg^2+^ is colored in pink. All the structural figures were generated using PyMOL (http://www.pymol.org).

G102 is located on the β-sheet of the NUS1 protein, directly binding to residues L240 and L242, as shown in the crystal structure of the human NgBR/DHDDS complex (Figure 2, the right side). The substitution of G102 with D102 forms an interaction between D102 and S133. Additionally, the side chain of D102 highly repels residues P238, V241 and F253, and thus the protein structure of the protein destroyed, impairing the protein function.

R290 is located on the contact surface of the NgBR/DHDDS complex. The coordination sphere of R290 includes residues from NgBR (E288 and K293), residues from DHDDS (D34 and E209), two IPEs, and three water molecules (Figure 2, the left side). When residue R290 is substituted, its interactions with E209, E288 and the three water molecules are lost. One of the three water molecules mediates and interaction between R290 and Mg^2+^. The interaction between residues R290 (NgBR) and E209 (DHDDS) contributes to the formation of the heterodimer. Therefore, the substitution of R290 with H290 has a great impact on the structure and function of the protein complex. In addition, the pathogenic variant p.R290H is homozygous, which is expected to cause a more dramatic reduction in NgBR activity, resulting in more severe clinical symptoms.

It is noteworthy that a heterozygous *NUS1* frameshift variant has been identified in 5 familial patients with different clinical features (6). Three patients had ID and absent seizures, and could be diagnosed with typical MRD55. One patient only presented with clonic seizures, and another one displayed Parkinsonism without epilepsy. Why these five patients presented with distinct phenotypes is unknown. We can only speculate that NUS1 pathogenic variants as well as epigenetic, and/or environmental factors interact with each other to produce the specific phenotypes.

The number of cases with the *NUS1* pathogenic variants is still too low to draw a genotype/phenotype correlation, especially given that the *NUS1* pathogenic variants can cause different diseases. The molecular mechanisms underlying the effects of the *NUS1* pathogenic variants in patients who presented with different diseases are unknown. Additional cases and functional studies are needed for a better understanding of *NUS1* gene and related diseases.

## Conclusion

In this study, we reported a Chinese MRD55 patient with detailed clinical phenotypes. The *de novo* variant of *NUS1* identified by WES was novel and was concluded to be responsible for the clinical presentation of the patient. With this novel pathogenic variant found in *NUS1*, we expanded the genotype spectrum associated with the MRD55 phenotype and provided the potential for a better understanding of the relationship between this rare disease and the pathogenic variants.

## Data Availability Statement

The raw data supporting the conclusions of this article will be made available by the authors, without undue reservation, to any qualified researcher.

## Ethics Statement

The studies involving human participants were reviewed and approved by The Ethics Committee of Shandong University Qilu Hospital (Qingdao). Written informed consent was obtained from the individual(s), and minor(s)' legal guardian/next of kin, for the publication of any potentially identifiable images or data included in this article.

## Author Contributions

PZ, JY, and QH cared for the patient, collected the clinical data of the patient and drafted the clinical portion of the manuscript. DC analyzed the results of WES and wrote the extra part of the manuscript. XY applied the clinical tests on patients. PL assisted the clinical test and recorded the clinical information. YZ and NY summarized characteristics of reported cases. PZ, DC, JY, and QH finally revised the manuscript. All authors read and approved the submitted version.

## Conflict of Interest

DC was employed by the company Running Gene Inc. The remaining authors declare that the research was conducted in the absence of any commercial or financial relationships that could be construed as a potential conflict of interest.

## Publisher's Note

All claims expressed in this article are solely those of the authors and do not necessarily represent those of their affiliated organizations, or those of the publisher, the editors and the reviewers. Any product that may be evaluated in this article, or claim that may be made by its manufacturer, is not guaranteed or endorsed by the publisher.
